# The biology and clinical potential of circulating tumor cells

**DOI:** 10.2478/raon-2024-0055

**Published:** 2024-09-15

**Authors:** Taja Lozar, Klara Gersak, Maja Cemazar, Cvetka Grasic Kuhar, Tanja Jesenko

**Affiliations:** Faculty of Medicine, University of Ljubljana, Ljubljana, Slovenia; Institute of Oncology Ljubljana, Ljubljana, Slovenia; General Hospital Izola, Izola, Slovenia; Faculty of Health Sciences, University of Primorska, Izola, Slovenia

Affiliations should be:

Taja Lozar^1^, Klara Gersak^1,2,3^, Maja Cemazar^2,4^, Cvetka Grasic Kuhar^2^, Tanja Jesenko^1,2^

^1^ Faculty of Medicine, University of Ljubljana, Ljubljana, Slovenia

^2^ Institute of Oncology Ljubljana, Ljubljana, Slovenia

^3^ General Hospital Izola, Izola, Slovenia

^4^ Faculty of Health Sciences, University of Primorska, Izola, Slovenia

